# The Alpha-Lipoic Acid Improves Survival and Prevents Irinotecan-Induced Inflammation and Intestinal Dysmotility in Mice

**DOI:** 10.3390/ph13110361

**Published:** 2020-11-03

**Authors:** Daniely V. S. Costa, Deiziane V. S. Costa, Caren N. S. Sousa, Angeline M. H. P. Silva, Ingridy S. Medeiros, Dainesy S. Martins, Conceição S. Martins, Ana L. V. Pequeno, Roberto C. P. Lima-Júnior, Pedro M. G. Soares, Silvânia M. M. Vasconcelos, Gerly A. C. Brito, Emmanuel P. Souza

**Affiliations:** 1 Department of Morphology, Faculty of Medicine, Federal University of Ceará, Fortaleza, CE 60430-170, Brazil; deiziane2009@gmail.com (D.V.S.C.); angelineholanda@hotmail.com (A.M.H.P.S.); josycristophe@hotmail.com (C.S.M.); analuizapequeno@hotmail.com (A.L.V.P.); pedrogsoares@hotmail.com (P.M.G.S.); gerlybrito@hotmail.com (G.A.C.B.); 2 Department of Physiology and Pharmacology, Faculty of Medicine, Federal University of Ceará, Fortaleza, CE 60430-275, Brazil; carensoarez@yahoo.com.br (C.N.S.S.); ingridy_s.m@hotmail.com (I.S.M.); dainy.santos@gmail.com (D.S.M.); robertocesarpljr@gmail.com (R.C.P.L.-J.); silvania_vasconcelos@yahoo.com.br (S.M.M.V.)

**Keywords:** irinotecan, inflammation, antioxidants, gut

## Abstract

Irinotecan, an anticancer drug, induces diarrhea and intestinal inflammation, resulting in an increase in the cost of care and in treatment delays. In this study, we investigated whether alpha-lipoic acid (α-LA) could improve irinotecan-mediated intestinal inflammation, diarrhea and dysmotility. Intestinal mucositis was induced by irinotecan injection (75 mg/kg, i.p., for 4 days) in Swiss mice. α-LA (50, 100 or 200 mg/kg, gavage) was administered daily 1 h before the injection of irinotecan. Duodenum tissues were obtained for inflammation and proliferation analysis. The outcomes: diarrhea, intestinal dysmotility, weight body loss and survival were evaluated. Compared with the control condition, irinotecan diminished (*p* < 0.05) intestinal villus height, caused a loss of crypt integrity and intense inflammatory cell infiltration, increased myeloperoxidase (MPO), IL-6 and IL-1β levels and decreased reduced glutathione (GSH) levels in duodenum segments and increased gastric retention and decreased liquid retention in the medial intestinal segment, resulting in increased intestinal transit, severe diarrhea and reduced survival (approximately 72%). Furthermore, α-LA (200 mg/kg) pretreatment ameliorated (*p* < 0.05) these irinotecan-induced effects. Our findings show that α-LA reduced irinotecan-induced inflammation, intestinal dysmotility and diarrhea, resulting in improved survival. α-LA may be a useful therapeutic agent for the treatment of gut dysmotility in patients with intestinal mucositis associated with irinotecan treatment.

## 1. Introduction

Irinotecan, a DNA topoisomerase I inhibitor, is an anticancer therapeutic widely used for the treatment of breast and colorectal cancer [1]. Irinotecan has considerably improved survival outcomes; however, many side effects, such as diarrhea, mucosal inflammation, vomiting and nausea, reduce the quality of life, cause life-threatening side effects, increase the cost of care, cause treatment delays and diminish compliance with treatment regimens, which may compromise long-term outcomes if chemotherapy is being administered for curative purposes [2].

Currently, treatment of the diarrhea induced by irinotecan has been based on dietary modification and administration of antidiarrheal drugs, such as loperamide, the somatostatin analog octreotide and deodorized tincture of opium. However, these current therapies often worsen existing chronic gastrointestinal symptoms or induce other side effects, including respiratory depression, arrhythmias, seizures, and neurotoxicity [2,3,4].

A variety of alternative therapies have been investigated, such as herbal medicines [4]. However, whether alpha-lipoic acid (α-LA; 1,2-ditiolane-3-pentanoic acid), also known as thioctic acid, can improve the outcomes of intestinal mucositis induced by irinotecan, such as diarrhea, intestinal inflammation and mortality, has not yet been investigated.

α-LA is a powerful natural antioxidant agent with a scavenging capacity for many reactive oxygen species produced by human cells from fatty acids and cysteine at very small concentrations [5,6,7]. Exogenous α-LA has been shown to have antioxidant effects similar to those of its endogenous isoform [6,8]. 

α-LA has been implicated as a potential coadjuvant treatment for diabetes [9], wound healing [10], endothelial dysfunction [11] and schizophrenia [12]. Furthermore, α-LA has been shown to inhibit tumor growth [13] and inflammation [14].

Given that irinotecan induces severe intestinal inflammation and diarrhea in humans and mice, we aimed to investigate whether α-LA can improve the outcome and intestinal damage induced by irinotecan in mice.

## 2. Results

### 2.1. α-LA Pretreatment Attenuates Irinotecan-Induced Histological Alterations in the Duodenum

**Figure 1:**
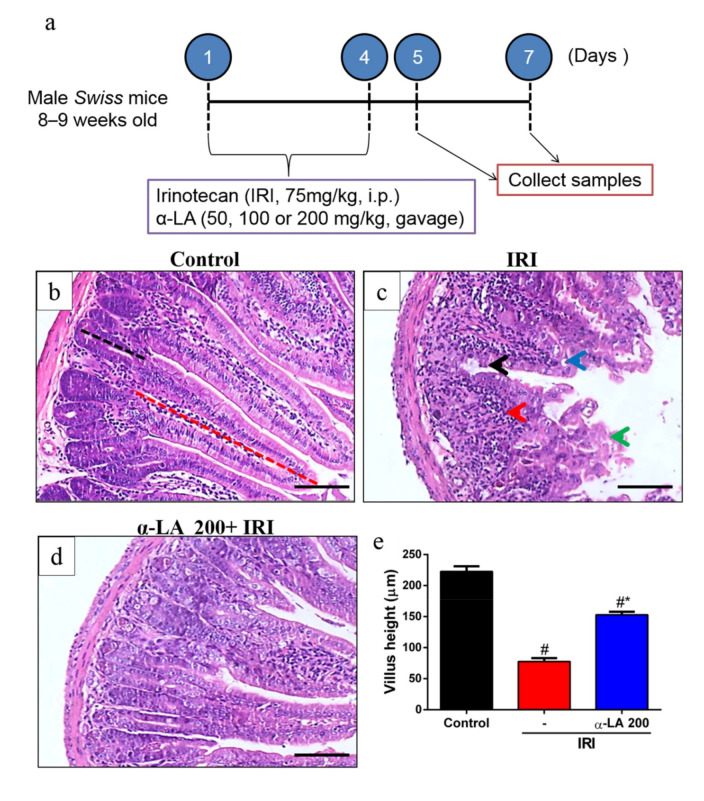
Alpha-lipoic acid (α-LA) pretreatment attenuates irinotecan-induced duodenum damage in mice. (**a**) Experimental timeline of intestinal mucositis induced by irinotecan and α-LA pretreatment. (**b**–**d**) Hematoxylin and eosin staining of the duodenum from control mice (**b**), showing intact villi (red dotted line) and crypts (black dotted line). Mice with irinotecan (IRI)-induced intestinal mucositis (IRI group) (**c**), characterized by an intense inflammatory cell infiltrate (red arrow), cell vacuolization (blue arrow), shortened villi (green arrow) and loss of intestinal crypt architecture (black arrow). Mice with irinotecan (IRI)-induced intestinal mucositis receiving α-LA pretreatment (**d**). Scale bars = 100μm. (**e**) Bars represent ± SEM of the villus height (ten villi per slide, *n* = 6) in µm. # represents *p* < 0.05 versus the control group, and * indicates *p* < 0.05 versus the IRI group. One-way ANOVA followed by Bonferroni’s test.

First, we evaluated whether α-LA pretreatment could prevent irinotecan-induced histological alterations in the duodenum, jejunum and ileum. Irinotecan induced severe intestinal injury characterized by intense inflammatory cell infiltrate, cell vacuolization, shortening of the villus and loss of intestinal crypt architecture (Figure 1c, Table 1), resulting in a maximum histological score (score = 3, Table 1). On the other hand, the control group showed intact villi and crypts (score = 0, Table 1, Figure 1b). However, α-LA pretreatment (200 mg/kg) was able to prevent these histological changes promoted by irinotecan only in the duodenum (score = 1, Table 1, Figure 1c). No significant difference was found between control mice and mice receiving α-LA (200 mg/kg) alone (Table 1).

Based on the histological score findings (Table 1), we selected 200 mg/kg as the optimal dose of α-LA to investigate its potential effects on other parameters. Of note, α-LA pretreatment in a dose of 200 mg/kg led to low histological scores values than 100 mg/kg (Table 1), showing that 200 mg/kg was more effective to prevent the intestinal damage induced by irinotecan.

In the villus height analysis, we found that compared with the control condition, irinotecan markedly decreased duodenum villus height (*p* < 0.05, Figure 1e). However, α-LA pretreatment increased duodenal villus height in mice with irinotecan-induced intestinal mucositis compared to mice that received only irinotecan (*p* < 0.05, Figure 1e).

### 2.2. α-LA Pretreatment Decreases Irinotecan-Induced Proliferation Inhibition in Duodenum Crypts

**Figure 2:**
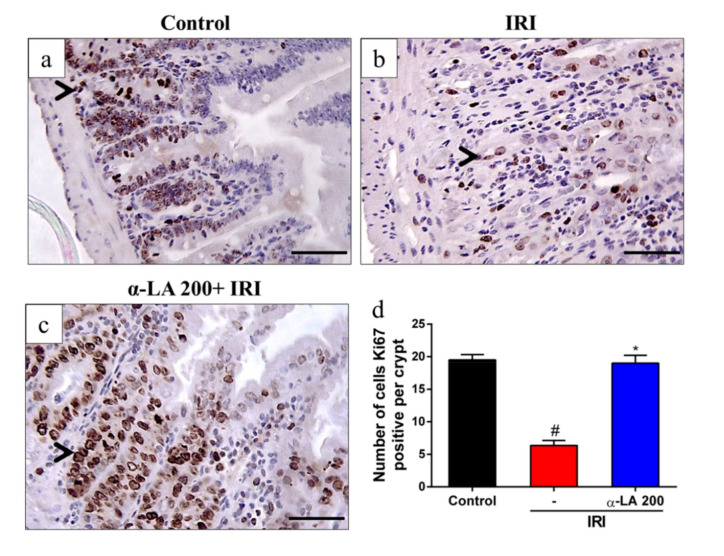
α-LA pretreatment decreases irinotecan-induced proliferation inhibition in the duodenum crypts in mice. (**a**–**c**) Representative immunohistochemistry staining for Ki67, a marker of proliferation, in sections of the duodenum of the control (**a**), IRI (**b**) and α-LA+IRI (**c**) groups. Scale bars = 50μm and magnification: 400×. Black arrows point to cells immunopositive for Ki67. (**d**) Bars represent ± SEM of the number of Ki67-positive cells per crypt (*n* = 4). # represents *p* < 0.05 versus the control group, and * indicates *p* < 0.05 versus the IRI group. One-way ANOVA followed by Bonferroni’s test.

We next tested whether α-LA pretreatment could prevent irinotecan-induced proliferation inhibition in duodenum crypts by performing an immunochemistry assay for Ki67, a proliferation marker. We observed intense immunostaining for Ki67 on the duodenal crypts of control group mice (Figure 2a). Compared with the control condition, irinotecan decreased (*p* < 0.05) the number of cells positive for Ki67 (Figure 2b,d) in duodenal crypts. However, α-LA pretreatment increased (*p* < 0.05) the immunostaining for Ki67 (Figure 2c,d) in mice with irinotecan-induced intestinal mucositis compared to mice receiving irinotecan alone.

### 2.3. α-LA Pretreatment Enhances Duodenal GSH Levels during Irinotecan-Induced Intestinal Mucositis in Mice

**Figure 3:**
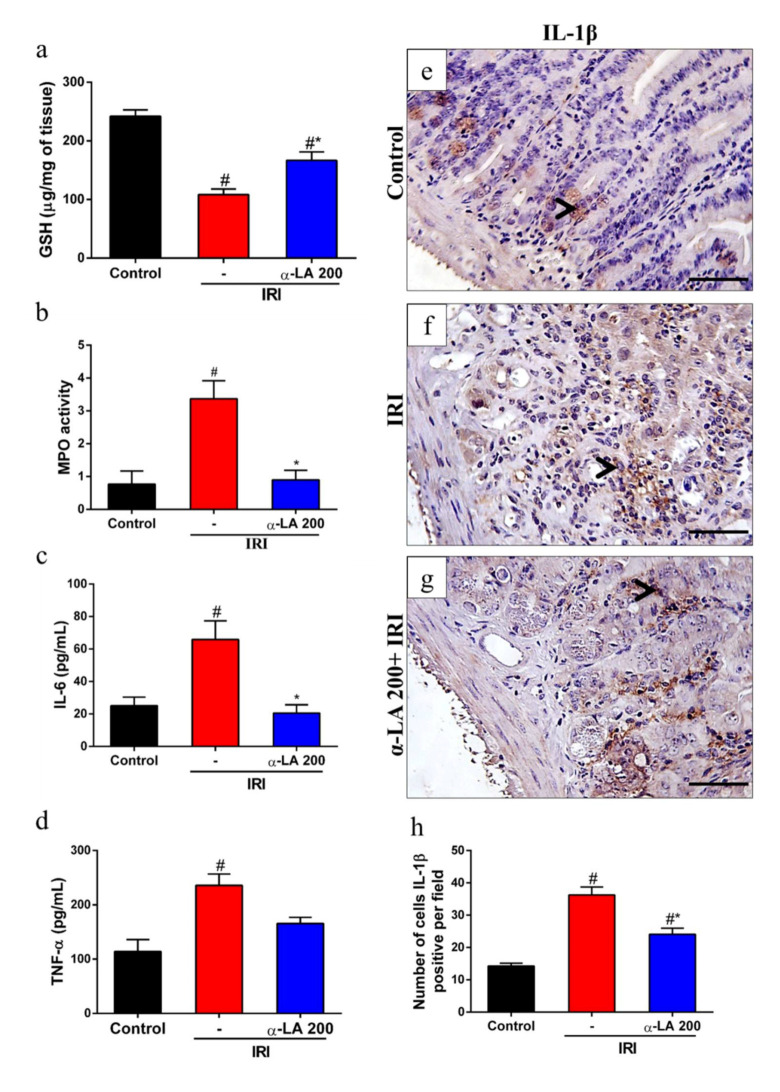
α-LA pretreatment decreases irinotecan-induced duodenum inflammation in mice. Levels of reduced glutathione (GSH) (**a**) and myeloperoxidase (MPO) (**b**) were evaluated by colorimetric assay, and levels of IL-6 (**c**) and TNF-α (**d**) were assayed by ELISA from duodenum samples of mice with irinotecan-induced intestinal mucositis that received α-LA treatment or no treatment. (**e**–**g**) Representative immunohistochemistry staining for IL-1β in sections of the duodenum of the control (**e**), IRI (**f**) and α-LA+IRI (**g**) groups. Black arrows point to IL-1β-positive cells. Scale bars = 50μm and magnification: 400×. (**h**) Bars represent ± SEM of the number of IL-1β-positive cells per field (*n* = 4). # represents *p* < 0.05 versus the control group, and * indicates *p* < 0.05 versus the IRI group. One-way ANOVA followed by Bonferroni’s test.

Given that α-LA is an antioxidant, we investigated whether α-LA affects the levels of GSH during intestinal mucositis induced by irinotecan. We found that irinotecan potentially decreased the levels of GSH in duodenum tissues compared to the control condition. On the other hand, α-LA increased the levels of this antioxidant in the duodenum of mice with irinotecan-induced intestinal mucositis compared to mice that received irinotecan alone (*p* < 0.05, Figure 3a).

### 2.4. α-LA Pretreatment Diminishes Irinotecan-Induced Duodenum Inflammation in Mice

Next, we assessed the potential role of α-LA treatment in the modulation of the inflammatory response during irinotecan-induced intestinal mucositis by measuring the levels of MPO activity, IL-6, TNF-α and IL-1β in duodenal samples. We found that irinotecan enhanced (*p* < 0.05) the levels of MPO activity (Figure 3b), suggesting increased neutrophil recruitment, and IL-6 (Figure 3c) and TNF-α expression (Figure 3d). In addition, we observed that irinotecan induced an increase in the number of positive cells for IL-1β in the intestinal epithelium and propria lamina of the duodenum of mice with intestinal mucositis induced by irinotecan compared to the control group (*p* < 0.05, Figure 3e,f). On the other hand, α-LA pretreatment decreased the expression of these inflammatory mediators (Figure 3b–f).

### 2.5. α-LA Pretreatment Prevents Irinotecan-Induced Diarrhea and Intestinal Dysmotility in Mice

**Figure 4:**
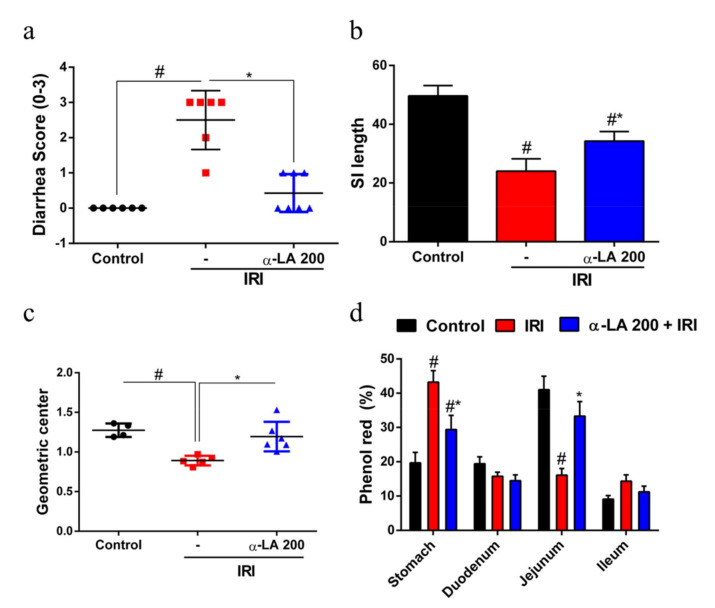
α-LA treatment prevents irinotecan-induced diarrhea and intestinal dysmotility in mice. (**a**) Diarrhea score on day 7 of the experimental protocol (0, normal; 1, slight; 2, moderate; 3, severe diarrhea). The results are expressed as the mean ± SEM (*n* = 6 per group). (**b**) Bars represent the mean ± SEM of the small intestine (SI) length (*n* = 6 per group). (**c**) and (**d**) indicate the relative distribution (geometric center) and percentage of phenol red in stomach and small intestine segments of mice with irinotecan-induced intestinal mucositis that received α-LA pretreatment or no pretreatment. The results are expressed as the mean ± SEM (*n* = 6 per group). # represents *p* < 0.05 versus the control group, and * indicates *p* < 0.05 versus the IRI group. One-way ANOVA followed by Bonferroni’s test.

In the evaluation of diarrhea at day 7 of the experimental protocol, we found that the irinotecan-treated group showed severe diarrhea (score = 3, *p* < 0.05) compared with the control group (score = 0), whereas α-LA pretreatment notably reduced (*p* < 0.05) irinotecan-induced diarrhea (Figure 4a).

In the analysis of small intestine length, we found that the length of the small intestine of mice that received irinotecan was decreased compared with control mice (*p* < 0.05). On the other hand, α-LA pretreatment increased the small intestine length in mice with irinotecan-induced intestinal mucositis (*p* < 0.05, Figure 4b).

Next, to explore whether α-LA pretreatment could prevent irinotecan-induced intestinal dysmotility, we measured gastrointestinal transit by examining the distribution of an orally administered phenol red dye along the gastrointestinal tract. We found that irinotecan changed (*p* < 0.05) the distribution of phenol red along the gastrointestinal tract, resulting in a significant reduction in gastric emptying and an increase in gastrointestinal transit compared with the control group (Figure 4c,d).

### 2.6. α-LA Pretreatment Diminishes Irinotecan-Induced Body Weight Loss in Mice

**Figure 5:**
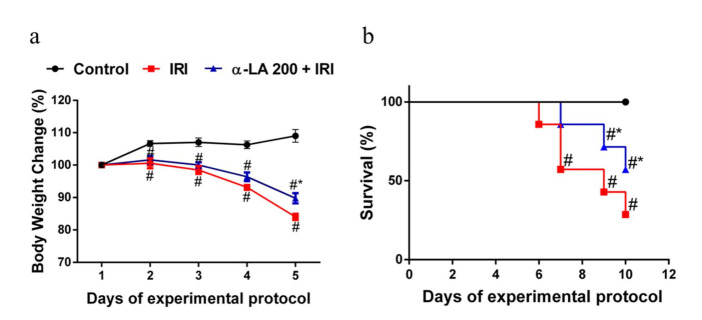
α-LA pretreatment ameliorates the outcomes (body weight loss and survival) of intestinal mucositis induced by irinotecan in mice. (**a**) Body weight was measured daily and is expressed as a percentage of initial body weight. The results are expressed as the mean ± SEM (*n* = 6 per group). # represents *p* < 0.05 versus the control group, and * indicates *p* < 0.05 versus the IRI group. Two-way ANOVA followed by Bonferroni’s test. (**b**) The survival curve represents the percentage of animals alive at the indicated time point after the first irinotecan dose. The mouse survival rate was evaluated using Kaplan–Meier curves. # represents *p* < 0.05 versus the control group, and * indicates *p* < 0.05 versus the IRI group. One-way ANOVA, Mantel–Cox-rank test.

As expected, compared with the control condition, irinotecan promoted notable (*p* < 0.05) body weight loss in mice, whereas α-LA pretreatment partially reduced (*p* < 0.05) the irinotecan-induced body weight loss (Figure 5a).

### 2.7. α-LA Pretreatment Improves the Survival of Mice with Irinotecan-Induced Intestinal Mucositis

To evaluate the effect of α-LA pretreatment on the survival of mice with intestinal mucositis induced by irinotecan, the mice were monitored for 10 days. As expected, compared with the control mice (no deaths), the mice that received only irinotecan exhibited a significantly (*p* < 0.05) decreased survival rate (57.0%) on day 7 of the experimental protocol, reaching 28% on day 10. α-LA pretreatment increased the survival rate of mice with irinotecan-induced intestinal mucositis (Figure 5b).

## 3. Discussion

In this study, we demonstrated that α-LA pretreatment decreased irinotecan-induced body weight loss by inhibiting diarrhea and decreasing duodenal inflammation, resulting in improved outcomes such as increased survival and decreased intestinal dysmotility. Together, these data indicate that α-LA pretreatment could be an effective and safe therapy for irinotecan-induced diarrhea.

The experimental model of irinotecan-induced intestinal mucositis was first described by Ikuno et al. [15] and adapted according for different animals according to characteristics that mimic the clinical symptoms presented by patients using this anticancer drug. Thus, the dose of irinotecan used in this study to induce intestinal mucositis in mice was 75 mg/kg, as used by previous studies [16,17,18]. This dose is able to mimic clinical symptoms such as diarrhea, reduced survival, weight loss, intestinal inflammation and changes in intestinal motility, as demonstrated in the present study.

Here, α-LA pretreatment decreased the weight loss induced by irinotecan. In agreement with our findings, α-LA promoted weight gain in rats subjected to early weaning stress [19] and in mice subjected to radiation-induced intestinal inflammation [20]. Several factors have been shown to contribute to the weight loss promoted by irinotecan, such as nausea, anorexia, loss of intestinal epithelial barrier integrity and diarrhea. In addition, accentuated weight loss induced by chemotherapy has been shown to contribute to a reduction in survival [21]. Of note, α-LA increased survival in mice with irinotecan-induced intestinal mucositis, as described by our findings.

α-LA pretreatment also decreased the intestinal damage and inflammatory cell infiltration induced by irinotecan in the duodenum. Similarly, α-LA-mediated effects were found in mice subjected to early weaning stress [19] and radiation-induced bowel inflammation [20]. In these mice, α-LA increased the villus length, as shown in our study. Because of these findings, the hypothesis was raised that villus length recovery could be associated with some of the effect of α-LA on intestinal epithelial cell proliferation. In our investigation of cell proliferation by Ki67 immunostaining in duodenal crypts, we found that α-LA reversed the effect of irinotecan on the proliferation of duodenal crypt epithelial cells, increasing Ki67 protein expression. In thyroid and breast cancer cell lines, α-LA inhibited the proliferation of these cells [13,22]. Therefore, these previous findings suggest that the effect of α-LA on the proliferation of intestinal crypt cells observed in the present study was an indirect mechanism. It is possible that the effect observed here was secondary to the decrease in inflammation promoted by α-LA during the intestinal mucositis induced by irinotecan.

α-LA is considered a potent antioxidant. As expected, α-LA decreased the consumption of GSH, a physiological antioxidant, in the duodenum of mice with intestinal mucositis induced by irinotecan. One of the mechanisms by which α-LA stimulates an increase in GSH is through the phosphorylation and translocation of nuclear transcription factor derived from erythroid 2 (Nrf2). Under normal conditions, Nrf2 is inactive in the cytoplasm and linked to Kelch-likeepoxycyclohexenone(ECH)- associated protein 1 (Keap1), which prevents its translocation to the nucleus. This factor is activated by changes in the redox state of the cell, such as increased ROS concentrations, oxidative damage byproducts and reduced antioxidant capacity [23]. In the nucleus, Nrf2 interacts with antioxidant response element to initiate the transcription of genes and proteins that contribute to neutralizing reactive oxygen species [24].

In addition, α-LA also reduced the recruitment of neutrophils in the duodenum during irinotecan-induced intestinal mucositis, as observed indirectly by the levels of MPO activity. Similarly, α-LA also reduced MPO levels in LPS-induced lung injury and trinitrobenzene sulfonic acid (TNBS)-induced colitis [25,26]. Neutrophils release enzymes such as metalloproteinases (MMP-9) and MPOs that contribute to tissue damage when released in large amounts by degrading tissue collagen [27]. During inflammation, proinflammatory cytokines, chemokines, and growth factors stimulate the recruitment of neutrophils to the lesion site. In this context, IL-1β has been shown to stimulate neutrophil migration [28]. This proinflammatory cytokine activates NFκB, promoting its translocation into the nucleus, which in turn stimulates the transcription of chemokines, adhesion molecules and proinflammatory cytokine genes, such as IL-6 and IL-1β [29].

Additionally, α-LA decreased the protein levels of IL-6 and IL-1β, but not TNF-α, in the duodenum tissues of mice with irinotecan-induced intestinal mucositis. In immunohistochemistry analysis, the decrease in IL-1β was observed mainly in the epithelium. As in the present study, α-LA decreased IL-1β and IL-6 in radiation-induced intestinal injury in mice [20]. IL-6 is considered a pleiotropic cytokine due to its proinflammatory and anti-inflammatory activities depending on its tissue levels and on the pathway activated. During inflammation, IL-6 can be released by intestinal epithelial cells, mast cells, glial cells and enteric neurons, stimulating the expression of IL-1α and TNF-α cytokines via NFκB [30]. In this context, it has previously been demonstrated that α-LA modulates the activation of NFκB in monocytes and reduces the activation of this transcription factor in radiation-induced intestinal injury in an experimental model [20,31]. Here, we did not demonstrate a decrease in NFκB activation directly; however, the decrease in IL-1β and IL-6 promoted by α-LA during the intestinal mucositis induced by irinotecan indirectly supports this hypothesis.

Furthermore, α-LA considerably improved the outcomes, diarrhea and intestinal dysmotility induced by irinotecan. As demonstrated here, α-LA has previously been shown to attenuate diarrhea in rats after early weaning from breastfeeding [19]. Here, these effects of α-LA on intestinal motility and diarrhea occurred in an acetylcholinesterase-independent manner, since α-LA did not prevent the inhibition of this enzyme induced by irinotecan in either the duodenum or jejunum (data not shown). Our results suggested that α-LA promoted a decrease in diarrhea and intestinal dysmotility through its anti-inflammatory effect on irinotecan-induced intestinal mucositis. In addition, it has also been shown in the present study that α-LA decreased the shortening of irinotecan-induced intestinal smooth muscle. Previous studies have shown that irinotecan promotes the shortening of the intestinal smooth muscle by promoting the hypercontractility of this intestinal muscle [17,32]. GI motility may be controlled by many factors, including neurotransmitters within the ENS and CNS, GI hormones, and various immune factors [33,34,35].

Because α-LA attenuated irinotecan-induced histological changes, inflammation, diarrhea, and dysmotility, it was hypothesized that α-LA would improve the survival of mice undergoing irinotecan-induced intestinal mucositis. Data from this study showed that α-LA was able to improve the survival of mice with irinotecan-induced intestinal mucositis.

## 4. Materials and Methods 

### 4.1. Mice

Seventy-six Swiss mice (8–9 weeks old) were housed in temperature-controlled rooms under 12-h light–dark cycles. The animals received water and food ad libitum. Surgical procedures and animal treatments were conducted in accordance with the Guidelines for Institutional and Animal Care and Use of the Federal University of Ceará, Brazil. All procedures involving animals were approved by the Federal University of Ceará Committee on the Ethical Treatment of Research Animals (Protocol No. 31/2016).

### 4.2. Induction of Experimental Intestinal Mucositis

Intestinal mucositis in mice was induced experimentally, as previously described [15,16]. Briefly, irinotecan (Bergamo, 75 mg/kg, i.p., once a day) was administered for 4 days, and on day 5 or 7, the mice were euthanized by rapid decapitation, as shown in Figure 1a. To study animal survival, the mice were observed as described previously up to day 10 following the first injection of irinotecan.

### 4.3. Experimental Groups

The mice were divided into six experimental groups: control (healthy mice that received only saline solution, i.p.), IRI (a positive control group of irinotecan-induced intestinal mucositis), α-LA +IRI (mice with irinotecan-induced intestinal mucositis that received 50, 100 or 200 mg/kg α-LA, gavage) and α-LA (healthy mice that received only 200 mg/kg, gavage). α-LA (Sigma-Aldrich,St. Louis, MO, USA, T5625) was administered 1 h before each irinotecan injection. Body weight was measured daily, and body weight changes were expressed as percentages of the baseline value.

The initial doses of α-LA (50, 100 or 200 mg/kg) were based on a previous study [36].

### 4.4. Histopathological Analysis

Intestine tissue samples (duodenum, jejunum and ileum) were fixed in 10% neutral buffered formalin, dehydrated and embedded in paraffin. Sections (5 µm thick) were obtained for hematoxylin and eosin (H&E) staining and for subsequent examination using light microscopy (200×). The lengths of the intestinal villi (8 to 10 villi per slide; 6 mice per group) were determined using ImageJ 1.4 (NIH, Bethesda, MD, USA). Mucosa injury was assessed using a modification of the histopathological scoring system described previously [37].

### 4.5. GSH (a Non-Protein Sulfhydryl) Measurement 

Duodenum tissue samples were collected on day 5 and homogenized in 1 mL of 0.02 M EDTA to measure glutathione (GSH) levels as described previously [38]. Aliquots of 200 μL of homogenate were added to 160 μL of distilled water and 40 μL of 50% trichloroacetic acid (TCA) to precipitate proteins. Then, the samples were centrifuged (3000 rpm, 15 min, 4 °C), 200 μL of supernatant was added to 400 μL of 0.4 M Tris (pH 8.9) and 10 μL of DTNB, and the mixture agitated for 3 min immediately before reading. The absorbance was measured at 412 nm. The results were expressed as μg of GSH per mg of tissue.

### 4.6. MPO Measurement

Myeloperoxidase (MPO) is an enzyme that is found most abundantly in azurophilic granules of neutrophils. It was measured in the present study as a neutrophil marker in duodenum segments by following a previously described colorimetric method [39]. Briefly, duodenum samples were homogenized in hexadecyltrimethyl-ammonium bromide buffer (50 mg of tissue per mL). The homogenates were then centrifuged at 3000× *g* for 15 min at 4 °C. MPO activity was evaluated by measuring the change in absorbance at 450 nm using a reading solution (5 mg of *o*-dianisidine, 15 μL of 1% H_2_O_2_, 3 mL of phosphate buffer and 27 mL of H_2_O). The change in absorbance was recorded and plotted on a standard curve of neutrophil density. The obtained data were expressed as MPO activity (units of MPO per mg of tissue).

### 4.7. Analysis of TNF-α and IL-6 Levels 

The duodenum samples were collected and homogenized in radioimmunoprecitation assay (RIPA) buffer to evaluate the levels of TNF-α and IL-6. These cytokines were measured by using a commercial ELISA kit (R&D Systems, Minneapolis, MN, USA) according to the manufacturer’s instructions. The levels of TNF-α and IL-6 in the duodenum were expressed as picograms per milliliter.

### 4.8. Immunohistochemistry

Sections (4 µm) were prepared from paraffin-embedded intestinal tissues. After deparaffinization, antigens were recovered with citrate buffer (pH 6.0) for 20 min at 95 °C. Endogenous peroxidase was blocked with 3% H_2_O_2_. The sections were then incubated with Ki67 (1:100, Abcam, Cambridge, MA, USA) and IL-1β (1:200, Santa Cruz Biotechnology, Dallas, TX, USA) antibodies diluted in antibody diluent (DAKO) for 1 h. Afterwards, the sections were incubated for 30 min with polymer (K4061, DAKO). The antibody binding sites were visualized by incubating with a diaminobenzidine (DAB)–H_2_O_2_ (DAKO) solution.

### 4.9. Diarrhea Assessment

Diarrhea was observed on day 7 of the experimental protocol (Figure 1a) to analyze delayed-onset diarrhea. The severity of diarrhea was scored as previously described [40]: 0 (normal), normal stool or absent; 1 (slight), slightly wet and soft stool; 2 (moderate), wet and unformed stool with moderate perianal staining of the coat; and 3 (severe) watery stool with severe perianal staining of the coat.

### 4.10. Gastric Emptying and Intestinal Transit

Gastric emptying and intestinal transit were measured on day 7 of the experimental protocol using a modified technique previously described [41]. Mice were deprived of food with free access to water for 18 h. Then, 0.3 mL of phenol red solution (0.75 mg/mL in 5% glucose) was administered by gavage to each mouse. After 20 min, the animals were euthanized, gastrointestinal tissues (stomach and small intestine) were collected, and their lengths were measured. The small intestine was divided into three segments: duodenum (40% of total small intestine length), jejunum (30% of total small intestine length) and ileum (30% of total small intestine length). Each gastrointestinal segment was placed in 15 mL tubes containing 10 mL of 0.1 N NaOH solution. The samples were cut into small pieces, mechanically homogenized and centrifuged (2800 rpm, 4 °C, 10 min). Then, 50 μL of 20% TCA was added to 1 mL of the supernatant to precipitate proteins from the homogenate, and the sample was centrifuged (2800 rpm, 4 °C, 20 min). Finally, 75 μL of each sample was added to 96-well plates, and 100 μL of 0.5 N NaOH was added to each well. The absorbance of the samples was analyzed using an ELISA reader (EPOCH, Winooski, VT, USA) at 570 nm. The fractional dye retention was expressed as a percentage, according to the following equation: Gastric dye retention = amount of phenol red recovered in stomach/total amount of phenol red recovered from two segments (stomach and small intestine). Intestinal transit was calculated for each intestinal segment by dividing the amount of phenol red recovered from a given segment by the amount of phenol red recovered from all three segments and was expressed as a percentage.

### 4.11. Statistical Analysis

All data were analyzed using GraphPad Prism 6 software (GraphPad Software). Data are presented as the mean ± standard error of the mean (SEM) or as medians when appropriate. Student’s *t* test or one or two-way analysis of variance (ANOVA) followed by the Bonferroni test was used to compare means, and the Kruskal–Wallis and Dunn tests were used to compare medians. *p* < 0.05 was considered significant.

## 5. Conclusions

**Figure 6:**
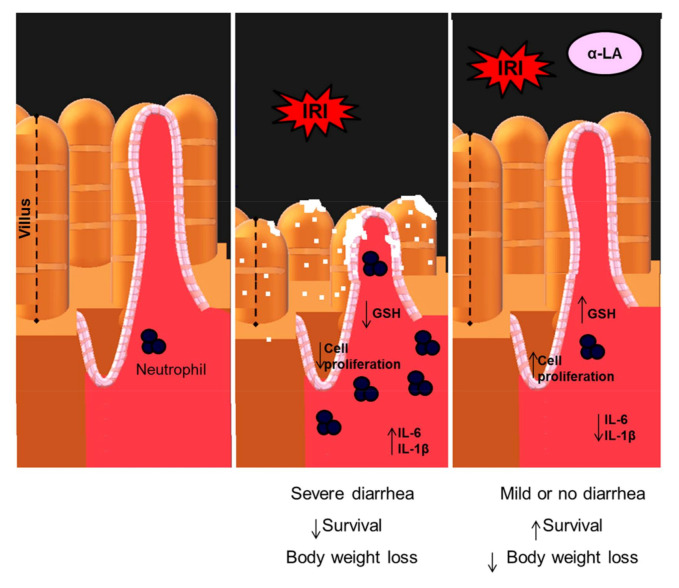
Proposed model of action of α-LA in the intestinal mucositis induced by irinotecan. Irinotecan (IRI) decreases the proliferation of intestinal crypt epithelial cells, promoting their breakdown and decreasing the intestinal villi. Abnormalities in these structures are potentiated by the decrease in GSH and the increase in IL-6 and IL-1β, which subsequently recruit neutrophils resulting in severe diarrhea, body weight loss and reduction in survival. Whereas α-LA prevents these irinotecan-induced changes by increasing the proliferation of crypt epithelial cells and inhibiting the synthesis of IL-1β and IL-6 resulting in the decrease in weight loss and the improvement in diarrhea and survival of animals with intestinal mucositis induced by irinotecan.

Finally, this study showed that α-LA prevents intestinal damage induced by irinotecan by acting as an antioxidant (increasing GSH) and inflammation inhibitor (decreasing recruitment of neutrophils and levels of IL-1β and IL-6), promoting proliferation of intestinal crypt epithelial cells and increase in the intestinal villi. Collectively, these effects of α-LA contributed to the decrease in body weight loss, inhibition of diarrhea and the improvement in the survival of mice with intestinal mucositis induced by irinotecan (Figure 6). α-LA may be a useful therapeutic agent for the treatment of gut dysmotility in patients with intestinal mucositis associated with irinotecan treatment. However, more investigations are needed for its safety use in humans under treatment with irinotecan. 

## Figures and Tables

**Table 1:** Histological scores for intestinal mucositis.

Intestine Segments	Experimental Groups					
	Control	IRI	α-LA50+IRI	α-LA100+IRI	α-LA200+IRI	α-LA200
**Duodenum**	0 (0–0)	3 (3–3) #	2 (2–3) #	2 (1–3) *	1 (0–2) *	0 (0–0)
**Jejunum**	0 (0–0)	3 (3–3) #	3 (3–3) #	3 (1–3) #	3 (2–3) #	0 (0–0)
**Ileum**	0 (0–0)	3 (2–3) #	2 (0–3)	2 (1–3) #	2 (0–3) #	0 (0–0)

Data represent median values (and range) of scores from 0 to 3: **Score 0**, normal histological findings; **Score 1**, mucosa: villus blunting, loss of crypt architecture, sparse inflammatory cell infiltration, vacuolization and edema. Muscle layer: normal. **Score 2**, mucosa: villus blunting with fattened and vacuolated cells, crypt necrosis, intense inflammatory cell infiltration, vacuolization and edema. Muscle layer: normal. **Score 3**, mucosa: villus blunting with fattened and vacuolated cells, crypt necrosis, intense inflammatory cell infiltration, vacuolization and edema. Muscle layer: edema, vacuolization and neutrophilic infiltration. Data were analyzed by using Kruskal–Wallis and Dunn´s test (*n* = 6). # represents *p* < 0.05 versus control group and * indicates *p* < 0.05 versus IRI group.
